# Occupational Disease as the Bane of Workers’ Lives: A Chronological Review of the Literature and Study of Its Development in Slovakia. Part 1

**DOI:** 10.3390/ijerph18115910

**Published:** 2021-05-31

**Authors:** Miriama Piňosová, Miriam Andrejiova, Miroslav Badida, Marek Moravec

**Affiliations:** 1 Department of Process and Environmental Engineering, Technical University of Košice, 040 01 Košice, Slovakia; miroslav.badida@tuke.sk (M.B.); marek.moravec@tuke.sk (M.M.); 2 Department of Applied Mathematics and Informatics, Technical University of Košice, 040 01 Košice, Slovakia; miriam.andrejiova@tuke.sk

**Keywords:** chronology, timeline, occupational diseases, recommendation, time series, Slovakia

## Abstract

This article not only offers a chronological overview of the development of occupational medicine, but also offers a summary of occupational diseases recommended by the ILO and legislative decisions that have influenced how we approach assessment today. We consider that these areas form a whole in which they cannot function without each other and they would lose their relevance if the system was collapsed. By excluding even one part of it, we would find ourselves at the beginning of the era of occupational medicine, and a large number of employees would once again be exposed to conditions that previously led to considerable illness and mortality of employees. The article also examines legislation and the development of occupational diseases in Slovakia in the period 1997–2019. Using basic statistical methods and time series, a trend model for the time series of the development of the number of occupational diseases over the last 20 years is created. The modeling also includes a forecast for the development of the number of occupational diseases for the next 5 years. The model created shows a favorable, decreasing trend in the number of occupational diseases in Slovakia.

## 1. Introduction

Occupational medicine is unique among medical fields because it focuses on the interface of the workplace and health. A healthy working environment is very important for economic and social development at the global and national levels. The occurrence of occupational diseases is a very important indicator of the quality of working conditions and the working environment. The aim of occupational hygiene is to ensure safety, health and well-being in the workplace and also to evaluate, prevent and control the risks related to the performance of work. Important occupational health problems that need to be addressed at the global level include inherent chemical, biological, physical, ergonomic and psychosocial risks.

Health protection at work is a multidisciplinary and cross-sectoral area that needs to be seen in the context of a country’s history and development.

Occupational medicine has undergone a long and complex development. The history of its development has been studied previously [1,2,3,4,5,6,7,8].

The development of occupational diseases has been monitored and evaluated by a large number of authors [9,10,11,12,13,14,15,16]. In their articles, they presented retrospective studies that analyzed the structure, causes, occurrence and trends in the development of occupational diseases over a certain period of time in a given country.

In 2019, Bentham Science Publisher published an e-book *Introduction to Occupational Health Hazards*, in which it was stated that “The study of the cause-effect relationship of occupational diseases will contribute towards reducing cases of work-related disorders” [17]. The book *Environmental and Occupational Medicine* (2007) [18] offers information on the history, causes, prevention and treatment of occupational diseases. Quick J.C. and Tetrick L.E. (2011) point out in their guide that work-related stress, along with other factors, can affect job productivity, satisfaction, safety, absence from work, etc. [19]. Carder M. et al. (2015) published an overview of occupational disease reporting systems in EU countries participating in the Modernet consortium [20]. The evaluation of occupational diseases in the EU was addressed by Nikolson P.J. [21]. The global burden of occupational diseases was tracked by Lesley Rushton (2017) [22], who found major gaps in data on exposure to dangerous factors, especially in developing countries. Most emerging economies in Africa still face a huge challenge in the area of occupational health and safety [23]. In most European countries, occupational diseases are underreported. The extremely low Hungarian figures are not a reassuring sign, but rather an alarming sign [10]. A major objective of the EU is to ensure a safer working environment for European workers. To this end, the EU issues directives that Member States implement into national law [24]. In 2017, the Chinese government issued a National Plan for Preventive and Treatment Procedures at Work to further protect health. The plan focuses on the urgent need to promote health at work [25].

The beginnings of workplace psychology are strongly related to the name Münsterberg H. [26], who published the book *Psychology and Industrial Efficiency* in 1913. The pioneers in the psychology of business management include Taylor F.W. (1856–1915) [27], the founder of “scientific management”, as well as Gilbreth F.B. (1868–1924) and his wife Gilbreth L.M. (1878–1932) [28]. The sociocentric approach is associated with the name Mayo G.E. (1880–1949). Mayo helped lay the foundation for the human relations movement and was known for his industrial research including the “Hawthorne Studies” and his books *The Human Problems of an Industrialized Civilization* (1933) and *The Social Problems of an Industrial Civilization* (1945) [29]. He proved that social relationships and informal social groups in the workplace are important factors in the performance (and satisfaction) of workers.

The results of a labor market analysis published by Grafton Slovakia at the end of 2020 show that more than half of those employed consider their workload to be excessive. Up to 60% of Slovaks feel stress at work. According to statistics from Everest College in the U.S., up to 83% of employees experience stress in the workplace. In the UK, 79% of employees face work-related stress, according to the UK Workplace Stress Survey 2020. According to the parallel survey done by Grafton in the Czech Republic, up to 70% of Czechs experience stress at work. In Slovakia, 12% of employees are often stressed and 48% experience stress at times, but regularly. There is also positive stress, which works on the basis of adrenaline, is short-lived and is considered motivating. Twenty-four percent of respondents have this type of stress in Slovakia [30].

This article offers a chronological overview of the literature in the field of occupational diseases, from the first mention of lung disease in stonemasons and metalworkers (4th century BC) to the present day. The aim of the article is a systematic examination of the history of occupational diseases in the world. The article also addresses the initial monitoring of the development of the incidence of occupational diseases in Slovakia. Using the method of exponential smoothing, a prediction of the number of diseases in Slovakia is made for the next five years.

## 2. Materials and Methods

### 2.1. General Overview

The occurrence of occupational diseases and poisoning at work is one of the most important indicators in caring for the health of employees carrying out risky work. It reflects not only the state of primary prevention of clinical manifestations of occupational harm to health but also the efforts of specialized professional health services in their diagnosis and reporting [31,32].

The ILO Employment Injury Benefits Recommendation, 1964 (No. 121), defines occupational diseases in the following terms: “Each Member should, under prescribed conditions, regard diseases known to arise out of the exposure to substances and dangerous conditions in processes, trades or occupations as occupational diseases”. Under the Protocol of 2002 to the Occupational Safety and Health Convention, 1981 (No. 155), the term occupational disease covers “any disease contracted as a result of exposure to risk factors arising from work activity” [33]. According to WHO, an occupational disease is “Any disease contracted primarily as a result of exposure to risk factors arising from work activity.” [34]. Work-related diseases have multiple causes, where factors in the work environment may play a role, together with other risk factors, in the development of such diseases. An occupational illness (or disease) is defined by the Occupational Safety and Health Administration (OSHA) as “any abnormal condition or disorder, other than one resulting from an occupational injury, caused by exposure to factors associated with employment.” [35]. The European Agency for Safety and Health at Work (EU-OSHA) provides the definition that a work-related disease “is any illness caused or made worse by workplace factors” [36].

Occupational diseases are characterized by the fact that the causal relationship between the pollutant and the disease is clear and indisputable. Under Section 8(2)(a) of Act no 461/2003 on social insurance: “An occupational disease under this Law is a disease recognized by the competent health establishment, included in the list of occupational diseases set out in Annex 1, if it has arisen under the conditions set out in that Annex to an employer’s employee under Section 16 in the performance of work tasks or duties or in direct connection with the performance of work tasks or duties.” [37]. The list of occupational diseases in Slovakia contains 47 entries; in Table 1 we list selected items from the list of occupational diseases, namely those that we examined when analyzing the development of the number of occupational diseases in Slovakia from 1987 until 2019 (see Section 3.3.2). The SK ISCO-08 national classification of occupations issued by Decree of the Statistical Office of the Slovak Republic No. 286/2007 is fully compatible with the International Standard Classification of Occupations ISCO-08 as recommended by Commission Recommendation No 200/824/EC of 29.10.2009. The SK NACE Rev. 2 statistical classification of economic activities is designed for categorizing data on all work activities performed by economic operators. SK NACE Rev. 2 is issued by Decree of the Statistical Office of the Slovak Republic No. 306/2007, and it is fully compatible with the European classification for the Countries of the European Community established by Regulation (EC) No 1893/2006 of the European Parliament and of the Council of 20 December 2006). 

A complete treatment of the whole area of the protection of health at work can be found in European Framework Directive 89/391/EEC [38]. In the legislation of the Slovak Republic, the area of risk assessment in the workplace is specified in the Labour Code No. 311/2011 [39] and Act No. 355/2007 [40].

**Figure 1:**
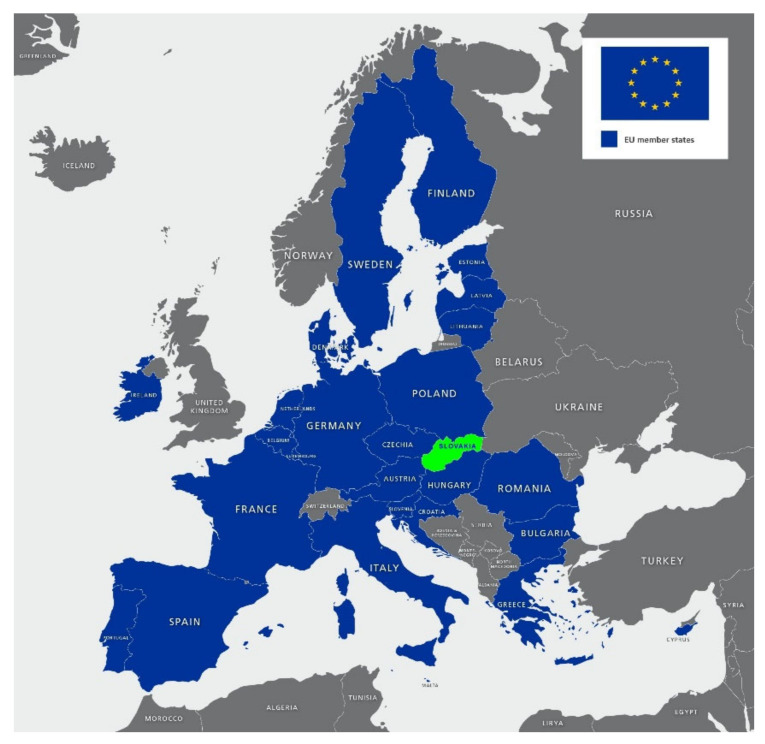
Location of the Slovakia in the EU [Base https://maproom.net/, accessed on 22 April 2021].

Slovakia (the Slovak Republic) is a landlocked country in Central Europe (Figure 1) with a total area of 49,035 km^2^. Approximately 5.45 million inhabitants live there, and the capital is Bratislava. Since 2004, it has been part of the European Union. Based on data from the Statistical Office of the Slovak Republic, there are 2.53 million working people registered in 2020.

### 2.2. Data Sources and Evaluation Methods

In preparing the chronological overview of the literature in the field of occupational diseases, and the occupational diseases as recommended by the International Labour Organization (ILO), we relied on electronic information sources, namely full-text databases (EBSCO, IEEE, Science Direct, PubMed), bibliographic and citation databases, digital libraries (Google Scholar, JSTOR, Semantic Scholar) and commercial research-sharing sites (ResearchGate). The literature review relevant to occupational disease is based on a thorough review of the work published in those sources. When we conducted the Review of the Literature on Occupational Disease, we categorized the data by subperiods for the 18th, 19th and 20th centuries with regard to the most important doctors, reformers, innovators and visionaries in the field in question. The chronology of progress in care for occupational health with regard to the above-mentioned is given in Table 2. The historical development of the ILO list of occupational diseases was based on processing the data available in the NORMLEX information system, which brings together information on international labor standards and also national labor and social security legislation. 

The evaluation of the development of the incidence of occupational diseases in Slovakia in the period 1997–2019 was based on data documented by the National Health Information Centre (NHIC), which belongs to the Ministry of Health of the Slovak Republic. The status and tasks of the NHIC are regulated by Act no. 153/2013 on the National Health Information System. At the international level, NHIC cooperates with the World Health Organization (WHO), Organisation for Economic Co-operation and Development (OECD) and EUROSTAT.

The basic methods of statistics and methods of analysis of time series were used to analyze and evaluate the number of occupational diseases in Slovakia. We use the time series to understand the sequence of factually and spatially comparable observations, which are clearly arranged in chronological order from the past to the present [41]. The time series forecast enables the quantitative estimates of future time series values that arise from prolonged future developments with a horizon h, provided that these developments do not change. In the article, we used the ExponenTial Smoothing (ETS) method to predict the development of the numbers of occupational diseases. The ETS is a forecasting method that predicts future values based on existing (historical) values using the Exponential Smoothing algorithm. The method is based on all previous observations, with their weight of older observations declining under the exponential function. Each model consists of three components: Error, Trend and Seasonal. The Error component can be described as “Additive = A” or “Multiplicative = M”. The Trend component can be described as “None = N”, “Additive = A”, “Additive damped = Ad”, “Multiplicative = M” or “Multiplicative damped = Md”. The Seasonal component can be “None = N”, “Additive = A” or “Multiplicative = M” [42]. There are 15 prediction models with additive errors and 15 models with multiplicative errors. Akaike’s Information Criterion (AIC) can be used to determine the best model. In general, the lower the AIC value, the better the model compares to a model with a higher AIC value. The time series prediction model is created in R using the package forecast.

## 3. Results and Discussion

### 3.1. Chronological Review of the Literature Occupational Diseases

**Figure 2:**
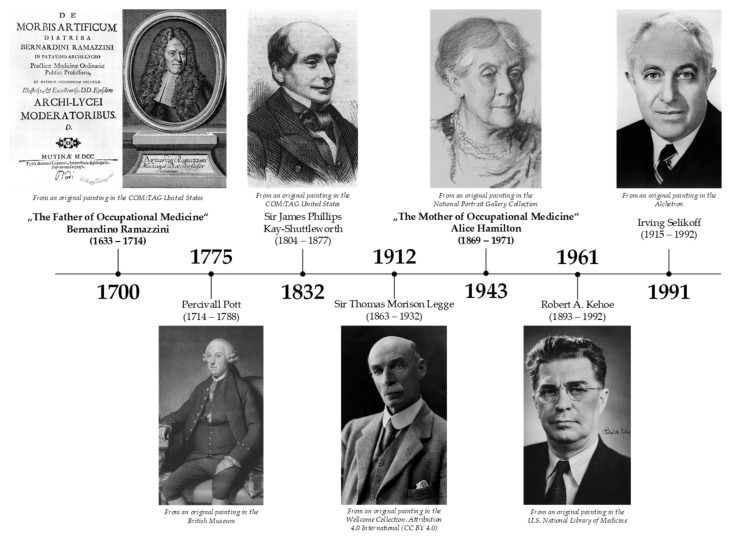
Timeline of the most important reformers of occupational medicine [Source: authors].

**Figure 3:**
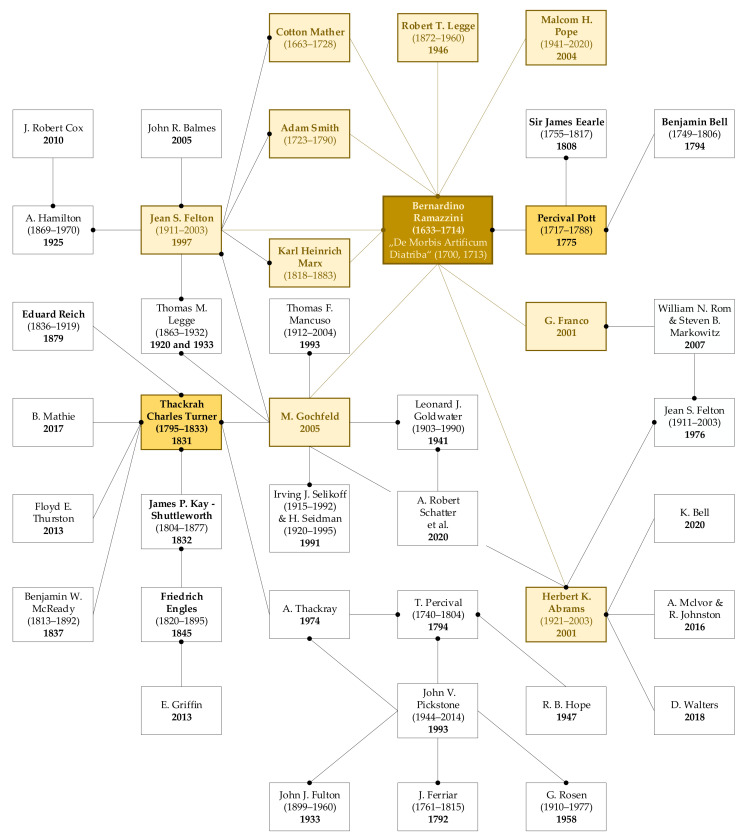
Map of articles in the field of occupational medicine starting with B. Ramazzini [Source: authors].

Occupation diseases have been with us since time immemorial and they have developed together with occupational medicine. As the nature of working activity has changed, new diseases have come along, and it has taken several decades for people to begin to associate them with the work they were doing. These diseases have been named “occupational diseases” [43]. Here, we present an overview of the most important scientists, doctors, reformers, visionaries in the field of occupational medicine, who shaped this field as we know it today (Figure 2 and Figure 3).

Already at the earliest historical stages of the development and life of society, many important representatives of medicine were interested in the social aspects of health care; for example, Hippocrates (460–370 BCE), who provides the first recorded mention of occupational diseases, describing dust in the lungs of stoneworkers and metalworkers; Aristotle (384–322 BCE); and Avicenna (CE 980–1037). “When you come to a patient’s house, you should ask him what sort of pains he has, what caused them, how many days he has been ill, whether the bowels are working and what sort of food he eats”, according to Hippocrates. The history of occupational medicine began to be written by Paracelsus (1493–1541) and Georg Agricola (1494–1555) in the 16th century, who particularly noticed the health problems of workers in manufactories and mines [4].

#### 3.1.1. The 18th Century 

Bernardino Ramazzini (†1714, Italian doctor) is considered to be “The Father of Occupational Medicine”. In 1700, he published the work “De Morbis Artificum Diatriba” [44] “Diseases of Workers”, in which he examined occupational diseases. This manuscript is considered to be a key work in the field of occupational medicine and has played an essential role in its development [45]. He described analytical and methodological approaches to the diagnosis and prevention of occupational diseases [46]. His successors, for example, Smith A., Marx K.H. and Mather C., and many other authors relied on this manuscript. He introduced two causes of occupational illnesses [5,7,45,47]. The first was the harmful effect of materials that employees handle at work. He found that many of them release harmful fumes and very fine particles into the air when processed, which adversely affects workers and causes serious illnesses. As a precautionary measure, he advised them to wash their hands and face frequently and even to stop working when they have difficulty breathing. He was of the opinion that insufficient ventilation and poor temperature control contribute significantly to the development of the disease. The second was attributed to intense and irregular movements, which are unnatural for proper posture. He claimed they caused such a disturbed physiological state of posture that serious occupational diseases could gradually develop. He supported rest, the need for exercise and a change in posture. 

Other important reformers of the 18th century include Lind J. (1753) [48], Scopoli G.A. (1761) [49], Pott P. (1775) [50], Parés y Franqués J. (1778) [51] and others.

#### 3.1.2. The 19th Century 

Charles Turner Thackrah (†1833, British doctor, reformer). In 1832, he drew attention to unsuitable working conditions at the Bean Ing Mills wool processing mill [52]. He described risks in various working sectors and pointed out that dust affecting the lungs of miners, metalworkers and other workers in dusty trades is linked to the development of tuberculosis. He warned of the long hours of child laborers in linen mills. In pottery, he recommended replacing lead glazes with others or completely changing working practices [53]. Several publications examining diseases of specific groups of workers already existed in the UK during this period. Pott P. (1775) [50,54], Bell B. (1794) [55] and Harrison E. (1827) [56] wrote about the incidence of cancer in chimney sweeps. A year after Thackrah’s research, Kay-Shuttleworth J.P. (1832) [57] published the book *The Moral and Physical Condition of the Working Classes Employed in the Cotton Manufacture in Manchester*. 

Benjamin William McCready (†1892, American doctor) [5] published “On the Influence of Trades, Professions, and Occupations in the United States, in the Production of Disease” (1837). This document is considered to be the first US study in the field of occupational medicine. 

Heinrich Hermann Robert Koch (†1910, German doctor) examined the bacterium *Bacillus anthracis*, which is the causative agent of anthrax. Based on his observations, he was able to determine the life cycle of anthrax bacteria and demonstrate a causal relationship between this microorganism and the development of the disease. In 1876, he published a study entitled “The Etiology of Anthrax Disease, Based on the Developmental History of Bacillus Anthracis” [58]. He also examined tuberculosis, cholera and other diseases [59]. He is considered the Father of Microbiology. In 1905, he was awarded the Nobel Prize in Physiology and Medicine for research and discovery in the treatment of tuberculosis. 

Louis Pasteur (†1895, a French doctor, chemist and biologist), known for his work on vaccines, was the first scientist to use live viruses in vaccination. He was working to create a vaccine against anthrax and rabies. Although Pasteur became famous because of his public speeches in 1881 and took credit for the creation of an anthrax vaccine [60], it is now believed that Jean Joseph Henri Toussaint (†1890, French veterinarian) was actually behind the creation of this vaccine. Pasteur’s nephew Adrien Loir (†1941, French bacteriologist) was aware of Toussaint’s work on vaccine development and therefore published a debate in 1938 entitled “À L’ombre de Pasteur”. Other important reformers of the 19th century include McCready B.W. (1837) [61], Chadwick E. (1842) [62], Engels F. (1845) [63], Virchow R. (1848) [64], Ireland G.H. (1886) [65] and others.

#### 3.1.3. The 20th Century 

Thomas Morison Legge (†1932, British doctor, inspector). He was the first factory inspector to focus on improving hygiene in industry [6]. In 1921, he participated in the Geneva Convention on the Prohibition of Painting Interiors with White Lead. Lead poisoning was the most common occupational disease at the time. Legge is also known for his work on anthrax. Anthrax disease often occurred in wool workers who were exposed to contaminated leather and wool. The mortality rate was high, with up to 1/4 fatality in all people suffering from pulmonary anthrax “wool-sorters’ disease” [4,66]. Legge summarized a wide range of industrial diseases, including cataracts, skin cancer, liver diseases and metal poisoning. A very important step in his life was the introduction of working medicine into the curriculum of the Faculty of Medicine. He was the author of several works, among the most important are (see Table 2). 

John Bertram Andrews (†1943, American economist) in 1909 became executive secretary of the American Association for Labor Legislation, which under his leadership was involved in drafting legislation in the area of labor law. He was the author of the books *Principles of Labor Legislation* (1916) [67], *Anthrax as an Occupational Disease* (1917) [68] and *History of Labor in the United States* (1918) [69]. 

Alice Hamilton (†1970, American doctor, industrial toxicology innovator). If Ramazzini is considered the Father of Occupational Medicine, Hamilton can be considered the “Mother.” She was a pioneer in the field of epidemiology of work and industrial hygiene. She began her long career in public health and workplace safety in 1908. She is the author of the first American guide on “Industrial Poisons in the United States” (1925) [70,71]. Her specialization was mainly industrial toxicology and health at work, which was related to her further publication “Industrial Toxicology” (1934), which she revised in cooperation with Hardy H.L. in 1949 [72]. Her research focused on the action of toxic substances (aniline dyes, mercury, carbon monoxide, tetraethyl lead, benzene, etc.) in the working environment; she investigated their effects on the body. She wrote about carbon monoxide poisoning among American steelworkers, mercury poisoning in milliners and the appearance of pulmonary tuberculosis in carvers in granite mills. Hamilton remained active in retirement, when she released her autobiography entitled *Exploring the Dangerous Trade* (1943) [73,74].

In 1942, a paper titled “Occupational Tumors and Allied Diseases” was published, which is considered the first medical textbook containing information about various types of cancers and the causes of their formation. In the introduction, the author pointed out the severity of chronic diseases [75]. This work was published by Wilhelm C. Hueper (†1979, a German-American doctor), who was a central figure in the field of occupational medicine and toxicology in the mid-20th century. He was one of the first scientists to attempt to educate the public about asbestos as a carcinogen in the working environment. In 1955, he published another one of his studies, “Silicosis, Asbestosis and Cancer of the Lung” [76]. He focused his attention on the effects of asbestos and coal tar. However, he erred in claiming that smoking contributes to occupational diseases to a lesser extent. This was before Selikoff I.J. et al. (1968) [77] provided evidence that in insulation workers there was a synergistic effect of smoking and working with asbestos, resulting in lung cancer. Throughout his career, Hueper sought to draw attention to the reluctance of businesses to acknowledge the fact that chemicals used in industry cause different types of cancer in employees. Twenty years later, he published the book *Chemical Carcinogenesis and Cancers* (1964) [78]. The culmination of his career was the book *Occupational and Environmental Cancers of the Urinary System* (1969) [79]. 

Robert A. Kehoe (†1970, an American toxicologist) was an expert in toxicokinetics. He focused his attention on monitoring the clinical manifestations of lead poisoning. From 1925 to 1965, he was a senior expert on lead in the US. In 1930, he became director of the Kettering Laboratory of Applied Physiology at the University of Cincinnati, the first university laboratory focused on toxicological problems in industry [80]. In 1953, he published research on “Experimental Studies on the Inhalation of Lead by Human Subjects” [81], in which he argued that the presence of lead in humans is normal and that exposure to it at low levels is not harmful. With this claim, he convinced the Ethyl Corporation that it did not have to worry about lead not only in the work area, but also in the environment, which contradicted the study by Hamilton [82,83]. 

Irving J. Selikoff (†1992, American doctor, researcher) created an extensive body of work documenting the high incidence of asbestos-related diseases during his four decades of work [84,85]. He found that workers exposed to asbestos also had scar tissue 30 years after their work ended. His research has put enormous pressure on OSHA. New York subsequently banned the use of sprayed asbestos during construction work in Manhattan. His study on insulation workers drew attention to the synergy between asbestos and tobacco smoking [77]. In 1966, he founded the Department of Environmental Medicine at Mount Sinai Hospital in New York. He was one of the founders of the Collegium Ramazzini, an independent international academy. 

Jean Spencer Felton (†2003, American academic, general practitioner). In 1958, he created the basis for a resident program in occupational medicine. In 1968, he became director of the health service in Los Angeles and later in Long Beach, where he researched the effects of asbestos. He has published countless books and articles on the history and practice of working medicine, [86,87,88,89,90,91,92,93] upon which researchers around the world rely to this day.

Thomas F. Mancuso (†2004, American doctor, epidemiologist) “changed the standards of occupational health protection” [94,95]. He published a series of articles highlighting the toxicological effects of materials such as asbestos, beryllium, chromium, cadmium, manganese, mercury and many other toxins. Working together with Hueper in 1951, he published research on the connection between chromium and lung cancer [96,97]. He revised these papers in 1997 and published them under the title “Chromium as an industrial carcinogen: Part I. and II.” [98,99]. In 1965, he was asked by the Atomic Energy Commission (AEC) to conduct a study on the effects of low-level radiation on a sample of half a million workers at the Hanford Nuclear Complex. He suggested that a long-term study was needed to accurately examine the cumulative effects. It showed that workers developed an increased risk of cancer caused by radiation levels that were considered safe at the time. Mancuso was later joined by doctor Stewart A. and statistician Kneale G. In 1977, they jointly published an article stating that workers at a nuclear weapons complex were dying of cancer induced by radiation at values that were well below the norm [100].

Kivimaki, Mika (*h*-index 125; 55,255), Shipley, Martin J. (*h*-index 92; 27,536), Ferrie, Jane (h-index 75; 11,391), Donhal, Kelley J. (*h*-index 39; 2755), Soteriades, Elpidoforos S. (*h*-index 22; 2640), Franco, G. (*h*-index 13; 394) and Balmes, John r. (*h*-index 12; 515) are all authors who can be considered 21st-century experts in this field, based on the *h*-index and the number of citations, not including self-citing articles.

### 3.2. Chronological Review of the List of Occupational Diseases Recommended by the International Labour Organization

The list of occupational diseases established by international and national legal systems plays an important role both in prevention and treatment and in compensation for workers’ diseases. This list is a set of officially recognized occupational diseases caused by exposure to danger during working activity. The list contains a definition of each occupational disease and it is based on basic legislation on occupational health and safety [119]. The first compensation schemes began to appear in the early 19th century. A number of factors (e.g., rapidly growing industrialization) contributed to their development. Since the introduction of occupational diseases as compensable diseases in the Act for the Compensation of Workers in Germany [120] (1871) subsequently in Switzerland (1877) and England (1880), legislation of this type was introduced in rapid succession across Europe [121]: Austria (1887), Norway (1895), Denmark (1897), Finland and Italy (1898) and France, Spain and Switzerland (1899). In the USA, the compensation scheme was only adopted in full in 1911 [8]. 

**Figure 4:**
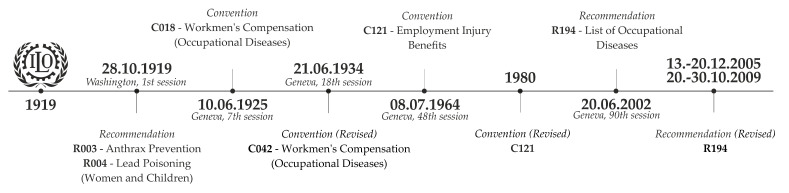
ILO Timeline [Source: authors].

The International Labour Organisation is a UN agency whose mandate is to promote social and economic justice by setting international labor standards. It was founded in 1919 after the end of the First World War in France. On 29 January 1919, the Commission on International Labour Legislation was established by the Peace Conference, with a view to drawing up the ILO Constitution. As a result of its work, a recommendation was made to create the ILO as a tripartite organization that would bring together representatives of member states’ governments, employers’ and workers’ representatives. The Labour Commission drafted a text entitled “Labour” [122], which became Part XIII of the Treaty of Versailles. In particular, the labor commission promoted the principles applicable to the conditions of work to be followed by the policy of the ILO Member States (Figure 4) and that were incorporated into Part XIII of the Treaty of Versailles—the Preamble to the ILO Constitution [123].

#### 3.2.1. The Era of Industrial Poisoning 

Following the ILO Conference on 29 October 1919 in Washington, anthrax and lead poisoning were declared occupational diseases. On 28.11.1919, the first two recommendations on the prevention of these diseases. R003—Anthrax Prevention Recommendation, 1919 (No. 3)—and R004—Lead Poisoning (Women and Children) Recommendation, 1919 (No. 4)—were adopted [124,125]. Although anthrax was first discovered in 1250 CE, the industrial revolution was responsible for the dangers created by it, and it was therefore the center of attention in those years. Maret (1752), Dym (1769) and Fournier (1769) made the first mentions of cutaneous anthrax [126,127]. Anthrax disease often occurred in English wool sorters and was known as “wool-sorters’ disease”; its mortality rate was high [66]. In addition to anthrax, the first industrial revolution brought with it a huge increase in demand for lead. Women and children were employed in all stages of the process, including very dangerous work in glazing ceramics, melting lead ores and the production of lead compounds [128]. This topic was examined by Ellenbog U. (1473), Thackrah C.T. (1832) and Kehoe, R.A. (1953) (see Table 2). After 1900, as a result of studies of industrial hygiene, many countries adopted legislation relating to the protection of workers’ health [66,129,130,131].

At its seventh session on 19 May 1925 in Geneva, after resolving to accept the proposal for workmen’s compensation, on 10.6.2015, the ILO adopted C018—Workmen’s Compensation (Occupational Diseases) Convention, 1925 (No. 18). Mercury poisoning was added to the list of diseases [132]. Like anthrax and lead, mercury experienced its biggest boom in the mid-19^th^ century with the development of industry. Hamilton A. (1943) [73] wrote about mercury poisoning in milliners. The production of hats at that time was dependent on mercury, and it was used in the form of a solution to accelerate the production of felt. An employee who entered a hat factory did not live for more than 3–5 years. Scopoli G.A. (1761) and Parés y Franqués J. (1778) wrote about mercury poisoning among miners as early as the second half of the 18th century (see Table 2). In 1934, C018 was amended and a new C042, Workmen’s Compensation (Occupational Diseases) Convention (Revised), 1934 (No. 42), was adopted. Another seven items were added to the list; see [133]. 

#### 3.2.2. Expansion of the ILO List of Occupational Diseases 

The list of occupational diseases with 10 items was used for 30 years unchanged until on 17 June 1964 it was revised, and a new C121—Employment Injury Benefits Convention, 1964 (No. 121) [134], was adopted. It continued to contain only a limited number of diseases, such as those identified by Stockhausen S. (1656) and Hoffmann F. (1716) and skin cancer as first described by Pott P. in 1775 [50,54,135] (see Table 2). 

The compensation system was very difficult to regulate. While the causal link in the case of poisoning was obvious, it was difficult to distinguish hearing loss or various bronchopulmonary and infectious diseases from those of the general population [94,136]. In the US in the early 20th century, it was the case that only hearing loss caused by immediate injury—explosion rather than gradually developed hearing loss—would be considered compensable [137]. In his book *Effects of Noise on Man*” [138], Kryter K.D. (1950) states that most published information on the effects of noise on humans is an “unsubstantiated expression” or is justified by “poorly designed experiments” [139]. The inclusion of noise-induced hearing loss in the list of diseases was therefore very difficult, and it only succeeded in 1980. This was also the case for asthma in the textile industry. Already in the early 18th century, Ramazzini B. described a special form of asthma in those who processed cotton, flax and hemp. He said the dust he observed while processing them “causes workers to cough constantly.” While many authors during the 19th and early 20th centuries were describing respiratory manifestations of occupational diseases in textile factories with increasing frequency, in the U.S., these diseases remained unnoticed until the mid-20th century when Schilling R.S.F. (1956) published the study “Byssinosis in Cotton and other Textile Workers” [140].

In 1980, with the growing public awareness of occupational diseases, Convention C121 was revised [141]. It now reflected the lessons learned over the last 70 years. During this period, there had been several fundamental changes, not only in the structure of industry (transition from heavy industry to services) but also in changes to the workplace risks (use of new industrial chemicals) and compensation policy. The revised version of C121 extended the original list with not only seven more types of poisoning, but also respiratory diseases, skin and infectious diseases, and disturbances caused by physical factors, and several types of work-related cancer were added. These were subsequently incorporated into the various compensation systems of different states [136,142,143,144,145,146,147,148]. In general, the lists were designed to identify specific diseases for which there would be evidence of causalities with one or more specific exposures at the workplace. 

The Employment Injury Benefits Convention, 1964 (No. 121), has so far been approved by 24 countries around the world [141]. Many countries have their own equivalent of this convention. On 22 May 1990, the Commission of the European Communities in Brussels approved recommendation (90/326/ECC) [149] on the adoption of the European Schedule of Occupational Diseases, which was revised 13 years later on 19 September 2003 (2003/670/EC) [150]. This list was more comprehensive than the ILO C121 list. Already in 1990, the European List contained a further 24 diseases caused by chemicals not listed in Convention C121. It also contained nine causes of skin diseases, including skin cancer, and 10 diseases caused by physical factors, including eight musculoskeletal disorders. This situation prompted the ILO in 1990–1991 to agree to the addition of Annex 1 to Convention C121, taking into account all legislation and practice, the most significant extension being the introduction of a detailed description of the procedures for diagnosing, reporting and evaluating occupational diseases in order to compensate them [119]. Among other things, the ILO prepared a list of occupational diseases that considered the currently valid lists and national practice in 76 countries. However, Article 31 of Convention No. 121 provides for a specific procedure for amending the list of occupational diseases set out in Annex 1 by a minimum of a two-thirds majority. Due to the competing priorities of the tripartite parties, the revision of the list could not be placed on the agenda. At the 90th session of the International Labour Organisation Conference on 3 June 2002 in Geneva, the process of changing the notification, diagnosis and identification of occupational diseases for the purpose of compensating them was approved.

#### 3.2.3. Further Updates to the ILO List Appended to R194 

The adoption of these changes was helped by the drafting and adoption of a new list of R194 recommendations—Letter of Occupational Diseases Recommendation, 2002 (No. 194)—which entered into force on 20 December 2002. This resolution recommended a new format for the list of occupational diseases, consisting of three basic categories for diagnosing diseases: the causative agent of the disease (chemicals, biological agents, physical factors), diseases by target organ (respiratory tract, skin diseases, musculoskeletal disorders and behavioral disorders) and occupational diseases of the cancerous-type. Sixteen chemicals, two physical agents, four pulmonary disorders and one skin disease [151] were added to the list. The category of cancer-type occupational diseases consisted of 14 carcinogens, the classification criterion of which was the category 1 list of the International Agency for Research on Cancer. Musculoskeletal disorders are also listed with a general definition of work-related diseases. The category “other diseases” is a flexible category that includes diseases not listed elsewhere. 

Recommendation R194 emphasizes its role as a tool for notification, the introduction of preventive measures, the improvement of the compensation procedure and the identification of the causes of occupational diseases [151]. Moving the list of occupational diseases from the Compensation Convention (C121) to Recommendation R194 has provided greater flexibility in drawing up a more comprehensive list. At its 279th session (November 2000), the governing body of the ILO recommended that the International Labour Organisation, at its 90th session, consider the development of a new mechanism for regularly updating the list of occupational diseases [152].

R194 was revised through two tripartite meetings in 2005 and 2009 [153,154]. The managing authority of the ILO convened a meeting of experts on 13–20 November 2008 in Geneva to update the list of occupational diseases [153]. In preparing the meeting, the ILO analyzed the 50 most up-to-date national lists of occupational diseases, including the recommended European Schedule of Occupational Diseases 2003/670/EC, and prepared a questionnaire on 34 issues related to changes, replacement, addition and re-categorization of occupational diseases, etc. Eighty Member States responded to this, 17 of which indicated that their responses were prepared after consultation with employers’ and employees’ representatives. Although most of the responses confirmed the proposed list with a few additional comments, some items, such as disease caused by radiofrequency radiation, cancer caused by formaldehyde and silica, or psychosomatic syndromes caused by bullying, were not accepted on the final list [155]. New entries included four diseases caused by chemicals, one caused by physical agents, five diseases caused by biological agents, two skin diseases, seven musculoskeletal disorders, two psychiatric and behavioral disorders and eight carcinogenic substances [156]. A further meeting on the revision of the Recommendation (No. 194) was held on 20–30 October 2009 involving 21 experts [154]. The new list was approved at the 307th meeting in March 2010. It replaced the previous list approved in 2002 as set out in Annex 1 to the Recommendation (No. 194). The new list included a total of 106 entries divided into three basic categories: disease agents (41 chemicals, 9 biological agents, 7 physical factors), target organ diseases (12 respiratory tract, 4 skin diseases, 8 musculoskeletal disorders and 2 behavioral disorders) and 21 cancer-type occupational diseases [33]. All revisions to those conventions or recommendations were influenced not only by the modernization of industry, but also by international organizations, and the European Union, and the development and revision of each state’s lists, which reflect the social, cultural and technological background of the time or country. 

### 3.3. Development of Occupational Diseases in Slovakia

In the second half of the 16th century and in the 17th century, a number of important Central European scholars appeared. There were humanitarians from various fields of science who came from Slovakia. Juraj (Georgius) Henisch (†1618, Slovak-German doctor, poet, polyhistor), born 24 April 1549 in Bardejov. He worked as a doctor in Augsburg, Germany. His scientific study “Arztney-Buch” [157,158] was one of the most popular medical works of the time. Karol Rayger (1641–1709) and Karol Oto Moller (1670–1747) made significant contributions to the development of medical sciences through their discoveries. In 1721, Prešov native doctor and pharmacist Ján Adam Raymann (1690–1770) entered world medical history with his research. Kežmarok doctor Daniel Perlitzi (1705–1778) prepared a proposal for the establishment of a university of medicine based in Banská Štiavnica. This proposal met with resistance from the Hungarian rulers of the time, who did not want to increase the educational level, even among children, of the Slovak nation they were ruling over, so it and many other attempts were unsuccessful.

The beginnings of care for health and safety care in our country date back to the 19th century, the Austro-Hungarian period. One of the pioneers of occupational medicine in Slovakia was František Xaver Schillinger (†1892, doctor) [159], who wrote a paper on cholera and first aid for miners. Gustáv Kazimír Zechenter-Laskomerský (†1908, doctor, writer, natural scientist) [160] studied the hygiene of the life and work of forest and mining workers and studied their diseases. Imre Tóth (†1928, doctor) [161] was the chief mining doctor in Banská Štiavnica. He wrote articles on the need to improve the environment and working environment of miners. In the fight against infectious and mining diseases, he contributed to reducing the incidence of diseases of lead miners, very widespread among metallurgical workers in Banská Štiavnica in the production of silver with lead. He proposed a range of measures to prevent this disease, which were directed to personal hygiene (handwashing, cleaning of workplaces and the use of respiratory protection). He also proposed technical measures to remove fumes from metallurgical furnaces. He also contributed to curbing the spread of tuberculosis and typhoid, and he publicly fought alcoholism. These authors understood health education as an integral part of medical activity.

Later, the Czechoslovak Republic (CSR) adopted the Act on the Compensation of Occupational Diseases on the basis of the Workmen’s Compensation (Occupational Diseases) Convention (No. 18) in 1932. In 1932, under the leadership of J. Teissinger the Occupational Diseases Advisory Board was created, which was transformed into the Occupational Medicine Advisory Board after 1942. After 1945, there was a strong development of occupational medicine institutes across the country. In 1949–1953, three institutes were established in Slovakia: in Bratislava, Martin and Košice. Their work was concerned with labor hygiene, the physiology of work and occupational diseases. 

In 1952, a Slovak branch of the society for occupational medicine was established within the J. E. Purkyně Czechoslovak Medical Society, which became independent in 1968 and still operates as an organizational component of the Slovak Medical Society. In the 1970s and 1980s, the issue of coal and ore mines came to the fore. In view of the occurrence of work-related diseases such as noise-related hearing loss, vibration diseases, dust on the lungs from dust containing silica (silicosis) and other respiratory diseases, these problems needed to be addressed without delay. The gradual reinforcement of the field with qualified personnel made it possible to develop and apply new methods of work and procedures in the field and in the laboratory. 

Industrial production in Slovakia was also focused on the extraction and processing of minerals, including coal and wood, iron and steel, heavy engineering and chemicals, posing high health risks to employees. These were large state-owned enterprises employing thousands of employees. With the adoption of Act no. 20/1966 on Care for Human Health, the requirements for the quality of the working environment and the conditions of work were further regulated and specified. Limits were set for harmful factors in the working environment. Directive 17/1970 of the Slovak Ministry of Health on the Assessment of Medical Fitness for Work laid down requirements for employers for the content, scope and frequency of medical preventive examinations and identified the categories of workers to undergo these examinations. In 1989, the Czechoslovak government ratified Convention (No. 155) from 1981 on Occupational Health and safety. In 1997, the National Reference Centre for Personal Exposure and Health Risk Assessment, today’s NHIC, was established.

#### 3.3.1. Legislation

The values of determining variables help to answer the question of to what extent the physical factors of work and the working environment pose a risk to the health of the employee or to what extent the measures taken are effective. Whether they are maintained or exceeded speaks not only of the level of risk, but also of the level of protection of employees’ health. Within the Slovak Republic, the basis for assessing the fulfillment of these requirements is the result of direct or indirect measurement and comparison with the values of determining variables laid down in decrees, government regulations and STN standards (taken from international standards).

The objectivity of physical factors of the environment and the working environment is monitored under Guideline OOFŽP-7674/2010 [162]. This guideline is used for the measurement of noise and vibration, daylight and artificial lighting, electromagnetic fields, the thermal-humidity microclimate and the other physical factors to be determined or evaluated at their place of occurrence. A complete treatment of the whole area of health protection at work can be found in European Framework Directive 89/391/EEC [38]. This Directive addresses the fact that employees may be exposed to dangerous environmental factors at the workplace during their working life. Since our legislation is currently harmonized with the EU, the notion of risk assessment and other concepts related to this procedure have also entered the legal norms of the Slovak Republic. In the legislation of the Slovak Republic, the area of risk assessment in the workplace is specified in Act no. 311/2011, the Labour Code [39], and in act No. 355/2007 [40]. 

Details of the factors of work and the working environment under the classification of works into categories are given in Annex 1 in Decree No 448/2007 [163]. The method of reporting and registering occupational disease and threatened occupational disease in the Slovak Republic is laid down by Act no. 355/2007 in Section 31b(1,2) [40]. The general principles of prevention and the basic conditions for ensuring health at work are laid down by Act no 124/2006 [164], and the requirements for the provision and use of personal protective equipment are laid down in Regulation No 395/2006 [165].

#### 3.3.2. Development of the Incidence of Occupational Disease in Slovakia from 1987 to 2019

The basic tasks of clinical occupational medicine and clinical toxicology in Slovakia include the comprehensive diagnosis, treatment and assessment of diseases arising in connection with adverse and health-damaging factors from work and the working environment. This includes reporting occupational diseases and threatened occupational disease. A total of 21,025 new occupational disease cases were reported in Slovakia between 1987 and 2019, based on data documented by the National Health Information Centre (NHIC).

**Figure 5:**
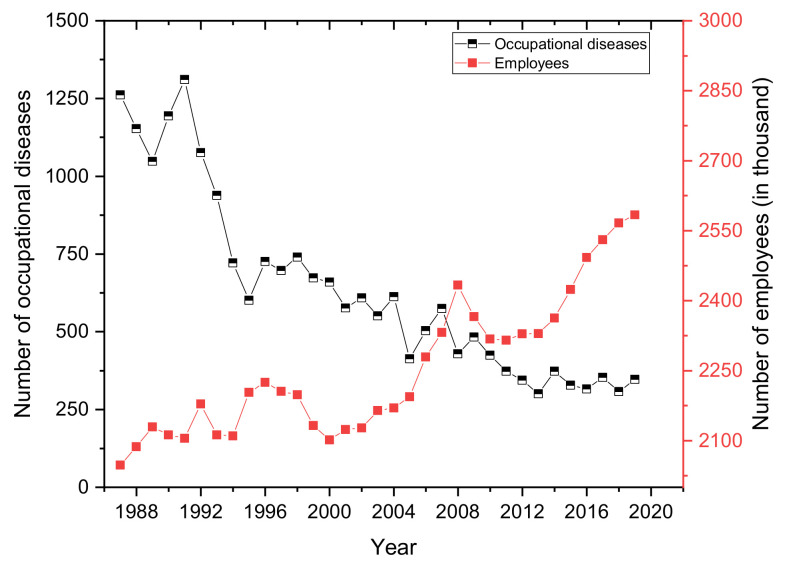
Development of the number of occupational diseases in Slovakia (1987–2019).

A graphical representation of the development of the number of occupational diseases in Slovakia for the period 1987 to 2019 is shown in Figure 5. The average annual number of recognized occupational diseases in the given period was almost 637. A significant decrease in the number of reported occupational diseases was recorded up to 1995, from 1262 reports (1987) with a slight increase of 1331 reports (1991) to 601 reports (1995). Between 1995–2019, the number of newly acquired occupational diseases decreased roughly in half with slight fluctuations, to 347 reports (2019), with an all-time low in 2013 (301 reports). In the long term, we are seeing a downward trend in the number of occupational diseases. The graph (Figure 5) shows the development of employment in Slovakia (1987–2019). The average annual value of the number of workers over the period is 2262.5 thousand persons.

The assessment of occupational diseases reported in the last 32 years (1987–2019) has seen a more pronounced decrease in the second half of the reference period (2003–2019), representing 49.76%, i.e., 6971 cases. 

**Figure 6:**
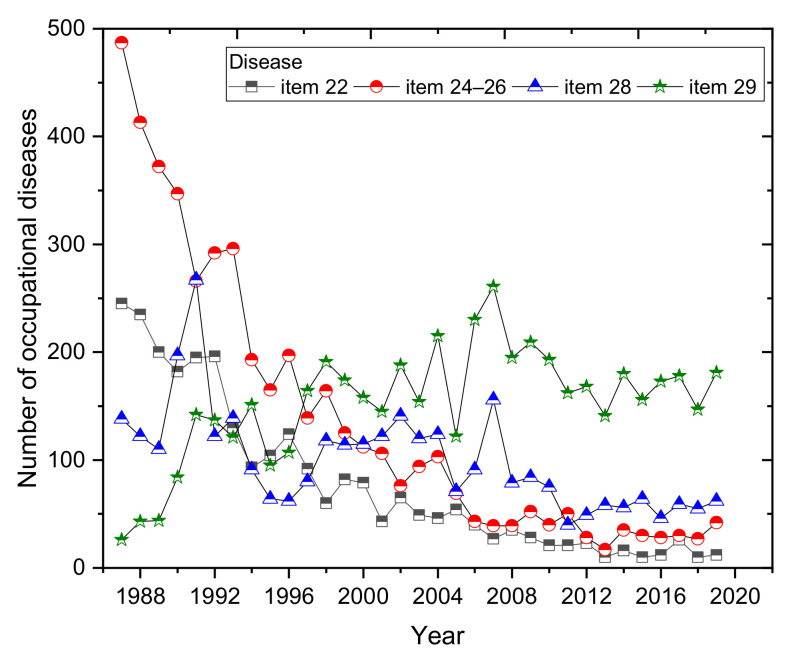
Development of the number of occupational disease cases in terms of selected types of disease (1987–2019).

**Figure 7:**
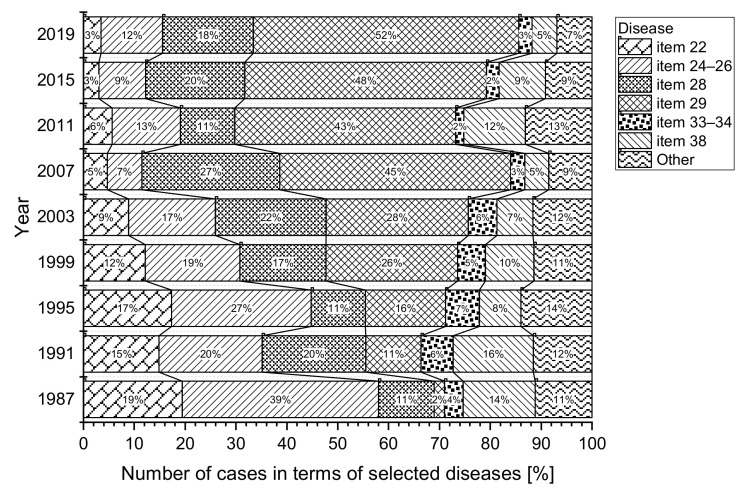
Percentage of the number of cases in terms of selected types of disease (1987–2019).

The most commonly reported occupational diseases include those listed in Table 3 (item 22, items 24–26, item 28, item 29, items 33–34 and item 38). Over the period considered, 19,142 new cases related to the diseases were reported, representing almost 91% of the total number of reported occupational diseases. The development of the number of occupational diseases in terms of selected diseases is shown in Figure 6. For the sake of clarity, only those diseases for which the average percentage of the total number of occupational diseases over a given period exceeded 10% are plotted in the graph. The percentage of selected occupational diseases out of the total number of reported cases in each year is shown in Figure 7. 

Compared to the first half of the period (1987–2002), we can see a decrease in almost all the selected types of occupational diseases in the second half (2003–2019) (Figure 6 and Table 3). The only exceptions are diseases affecting the musculoskeletal, vascular and nervous systems of employees exposed at work to prolonged excessive and one-sided loads on the upper limbs (item 29).

Despite a significant decreasing trend in the incidence of reported occupational diseases, limb disease from long-term, excessive and one-sided loads (item 29) is not developing very favorably (Figure 6). The annual incidence of reported diseases of the limbs from long-term excessive and one-sided loads on the limbs began to increase significantly from 1991. The largest number of reports (230 cases) was recorded in 2006, representing almost 46% of the total number of cases (504) in that year. In 2016, the proportion had increased to 55% (Figure 7). Compared to 1987, there was an increase of 885% in reported limb diseases in 2006 due to long-term excessive and one-sided loads. Between 2003 and 2019, we saw an increase of 55.58% in the incidence of occupational diseases, i.e., 3065 cases, overwhelmingly in women (item 29). 

Vibration occupational disease (item 28) has long been one of the most common occupational diseases in Slovakia. After limb disease from prolonged excessive and one-sided loads, vibration disease (with the exception of 2011, when noise-related hearing loss was temporarily in second place) has consistently come second among the numbers of annually reported occupational diseases in the last two decades. The high numbers in 1987–2007 gradually led to a significant decrease over 2008–2019, with the lowest incidence in 2011 (40 cases), and in the following years, the numbers have increased slightly (Figure 6). 

Between 2003 and 2019, there was a very significant decrease in the incidence of skin diseases (excluding skin cancer) and communicable skin diseases (item 22) compared to the previous period (1987–2002), by 79.29%, a decrease of 1685 cases (Table 3). Almost the same percentage decrease (79.57%) was also seen in cases of infectious diseases and parasitic diseases and diseases communicable from animals to humans (items 24–26). 

Noise-related hearing loss (item 38) is repeatedly in fourth or fifth place in the order of frequency of the number of cases of annually reported occupational diseases. The annual incidence of reported noise-related hearing damage decreased significantly between 1987 and 2008. In 2009–2014, a rise in these diseases was again noted, and they subsequently decreased in 2015 with slight fluctuations. The lowest incidence was recorded in 2008 and 2019, with 17 cases. Cancer-type occupation diseases listed under (items 21 and 23) were reported in 177 employees. The number of annual reports fell by 77.08% between 2002 and 2019, and a decrease of 111 cases (Table 3). The highest incidence was recorded in 1993; with 15 cases. The average annual number of reports was five cases. The average annual incidence of lung-related occupational diseases (items 33–34) is 27 cases, representing 3% of the total number of occupational diseases over the whole reporting period. In the case of (item 46), we can mention a negligible number of reported occupational diseases over the whole period under review (1987–2019), namely 37 cases. According to archive records, the disease was not diagnosed until 2003.

#### 3.3.3. Analysis of the Development of Occupational Diseases in Slovakia over the Last 20 Years

Available data show that a total of 8883 new cases of occupational diseases were reported in the last 20 years (from 2000 to 2019). The average annual number of cases of recognized occupational diseases in the given period is 444 cases. The trend in the incidence of occupational diseases in Slovakia is decreasing in nature. The average annual decrease in the number of occupational diseases is 16, representing an annual decrease of about 3%.

For example, the calculated dynamics in the number of occupational diseases show that in 2005, the number decreased by 200 cases compared to the previous year, representing a decrease of around 67.4%. On the other hand, there was an increase of 91 cases in 2006, representing an increase of around 22% in the number of occupational diseases compared to 2005. In 2019, 347 cases of occupational diseases were reported. This is 13.4% per 100,000 workers. Compared to the situation as of 31 December 2018, the number of reported occupational diseases increased by 39 cases (11.24%). Compared to 2000, there are 313 fewer cases of occupational diseases in 2019, almost 53% fewer cases than in 2000. 

When analyzing the number of occupational diseases, we selected three indicators (Table 4): the gender of workers (two subcategories), the age category (five subcategories) and the sectoral classification of economic activities (four subcategories). 

**Figure 8:**
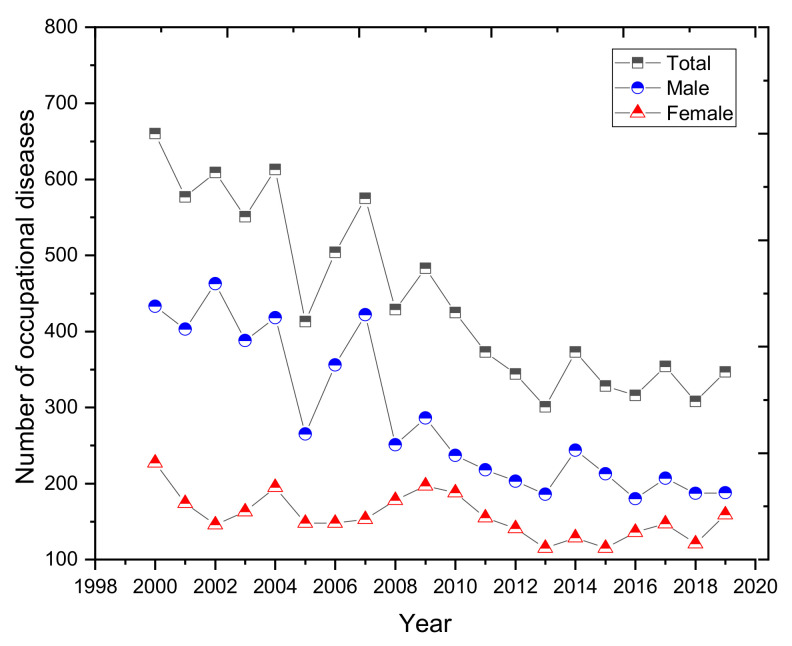
Development of the number of diseases in terms of the gender of workers (2000–2019).

A graphical representation of the development of the number of occupational diseases by workers’ gender is shown in Figure 8. Men are more heavily represented in the total number of diseases. Men were diagnosed with occupational diseases 1.8 times more often than women. In 2007, men were diagnosed with 422 cases of occupational diseases (as much as 73% of the total number of reported diseases), representing almost 2.8 times more cases than in women. The data show that over a period of 20 years we can see a significantly decreasing trend in the number of occupational diseases in men. 

**Figure 9:**
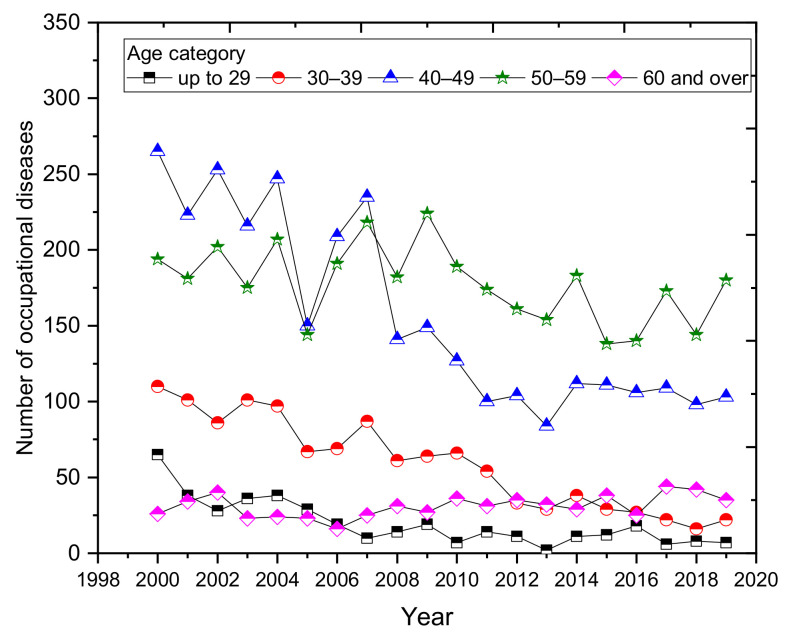
Development of the number of occupational diseases by age category (2000–2019).

Since 2008, the most commonly reported cases have been in the age group 50–59 years (Figure 9). The average representation of this age group in the total number of occupational diseases is almost 42%, compared with 52% in 2019. The second most common age category is the 40–49 category, for whom the average share of the total number of diseases diagnosed is almost 34%. In recent years, the number of reported cases in the over-60 category has increased slightly. On the other hand, the number of reported occupational diseases in the 30 to 39 age group is on a downward trend. A graphical representation of the development of the number of cases of diseases by age category is shown in Figure 9.

**Figure 10:**
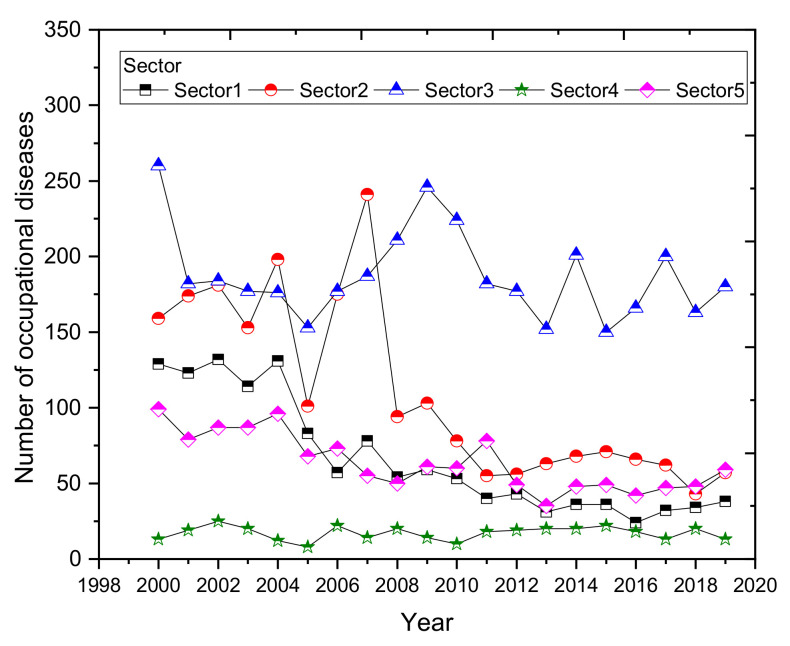
Development of the number of occupational diseases by economic activity sector (2000–2019).

A graphical representation of the development of the number of diseases by sector of economic activity is shown in Figure 10. The highest incidence of occupational diseases based on the sectoral classification of economic activities was in the industrial production sector (Sector 3). Over 20 years, 3748 cases were reported in the sector, representing 42.2% of the total number reported during the period. The lowest number of recognized occupational diseases in the period was in construction (Sector 4, 340 cases, 3.8% of the total number of diseases diagnosed). In 2007, the number of diseases from mining and quarrying professions (Sector 2) increased sharply. This was an increase of as much as 38% compared to the previous year and a 139% increase compared to 2005. In almost all sectors, we see a downward trend in the number of diseases. The only sector that maintains a constant trend is the construction sector (Sector 4). The average proportion of occupational diseases diagnosed in construction is 4%.

We used the ETS (ExponenTial Smoothing) method to determine the time-series model for the number of occupational diseases for the period 2000–2019 and the forecasts for the coming period. The resulting time-series prediction model consists of three components: Error, Trend and Seasonal. We have taken into account several models (Table 5) with different suitable combinations of the types of all three components. The ETS(M,A,N) model with multiplicative errors, additive trend and no seasonality represents Holt’s linear method with multiplicative errors. The ETS(A,A,N) model means Holt’s linear method with additive errors, ETS(A,N,N) means simple exponential smoothing with additive errors, etc. We compared the created models using the AIC criteria, with the best model being the model with the lowest AIC value.

**Figure 11:**
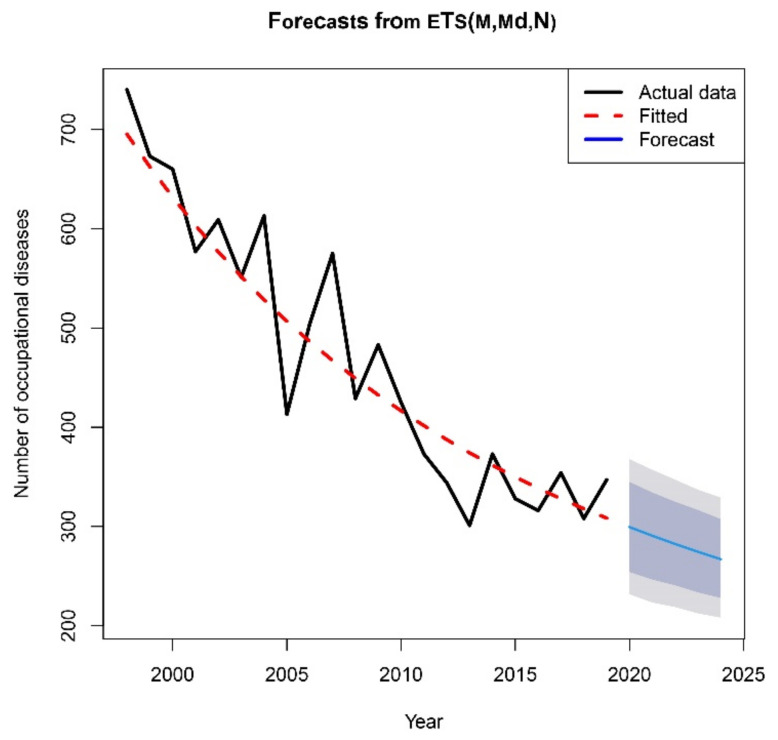
Development and forecast of the number of occupational diseases [Output: R].

It was found that the best model is in the form of ETS(M,Md,N), which means a damped trend (Md) with multiplicative errors (M) and no seasonality (N). The damping parameter is 0.97. A graphical representation of the original and equal time series obtained using the ETS method is shown in Figure 11. The graph shows a forecast for the development of the number of occupational diseases over the next five years. In addition to the forecast point estimate, prediction intervals are also created. The grey or blue area displays 95%, or 80% prediction intervals for forecasts obtained by the ETS(M,Md,N) model. 

The projection of the development of the number of occupational diseases in Slovakia over a period of 5 years obtained through the best model of ETS(M,Md,N) is shown in Table 6. 

We can state that the development of the number of occupational diseases diagnosed in Slovakia has been on a downward trend during 20 years of monitoring. This favorable trend may be related to a number of factors, including, in particular, increased responsibility of employers and employees who comply with the statutory principles of occupational health and safety.

## 4. Conclusions

During the nineteenth century, the essence of work underwent a major change. Production increased due to the increased efficiency and effectiveness of the means of production, while working conditions in factories, mines or workshops were often unfavorable. We are now at the beginning of the fourth industrial revolution, which is led by multinationals and information technology. Yet, even at this time, occupational diseases are a society-wide health problem with economic, social and labor-law aspects. It is estimated that up to 2 million people die from occupational disease per year, and up to 160 million are diagnosed with diseases that have been caused by the work being done [166].

This study provides a historical overview of the development of occupational diseases in the world and in Slovakia. The study also includes the development of the incidence of occupational diseases in Slovakia in the period 1997–2019, and it forecasts the development of the numbers for the next five years.

The results from the available data show that the trend in the number of occupational diseases in Slovakia is favorable; i.e., the number of cases of occupational diseases diagnosed is declining in the long term. This favorable development can also be attributed to the activities of regional public health authorities and occupational health services. Their activities include guiding the social and health prevention of diseases and harm to health from work by promoting national strategies, priorities and programs for the protection, promotion and development of the public health of employees. Through long-term improvement in the development of preventive occupational medicine and public health, various preventive measures, education of workers and raising awareness, these professions have contributed to the reduction of occupational diseases in Slovakia. 

In this paper (Part 1), we examined the historical development of occupational diseases and at the same time the development of the incidence of occupational diseases in Slovakia. In the next part of our research, we will specifically focus on a group of diseases from professions that arise when working in noisy or dusty environments, when working with vibrating tools, or when working long-term with one-sided loads. The aim of the research will be to identify, through appropriate statistical methods, the extent to which physical factors of work and the working environment, or other input variables (e.g., age, employment, general state of health of the working person), affect the development of an occupational disease. This research is of great importance for practice, since the occurrence of occupational diseases is one of the important indicators of the level of care for the health of employees and reflects the state of primary prevention of occupational diseases.

## Figures and Tables

**Table 1:** Selected occupational diseases.

Item	Name of Disease	Conditions under Which They Arise
21	Skin cancer diseases	In the manufacture, processing, use and transport of harmful substances causing skin cancer
22	Occupational dermatoses—Skin diseases apart from skin cancer and communicable skin diseases.	Exposure to the influence of chemical, physical (except ionizing radiation) and biological pollutants in the working environment and at work, causing occupational dermatosis
23	Diseases on pulmonary cancer from radioactive substances	When exposed to the stated hazard
24–26 ^1^	Infectious and parasitic diseases including tropical infectious diseases and parasitic diseases and diseases transmissible from animals to humans.	When exposed to the stated hazard
28	Vibration disease—Diseases of bones, joints, muscles, vessels, and nerves limbs caused at work with vibrating tools and device.	When in contact with sources of vibration
29	Diseases of bones, joints, tendons, and nerves of limbs from long-term, inordinate, one-sided workload.	When exposed to the stated hazard
33–34 ^2^	Pneumoconiosis	When exposed to the stated hazard
38	Hearing defect from noise by which loss hearing occurs, according to Fowler, with harm younger for those younger than 30 years at least 40%. Harm for those older than 30 years is then such that the presented level is increased by 1% every two years until 50 years of age of the harmed person, and since that time, loss of hearing must exceeded 50%.	When exposed to excessive noise
46	Tumor diseases emergent due to work with settled chemical carcinogens in damaged working environment and demonstrative in particular targeted organons, which are not included in this list.	When exposed to the risk of chemical carcinogenicity and a carcinogen is proven in the work environment, it is predominantly assessed by the National Commission for the Assessment of Occupational Diseases as the main causal factor in the development of a given cancer

^1^ (24) Diseases of communicable and parasitic illnesses apart from tropical communicable and parasitic diseases and illnesses communicable from animals to people. (25) Tropical communicable and parasitic diseases. (26) Illnesses communicable from animals to people directly or by means of communicants. ^2^ (33) Diseases from dusting of the lung with dust containing silicon oxide (silicosis, silicotuberculosis) including (miner pneumoconiosis). (34) Diseases from dusting of the lung with asbestos dust (Asbestosis). The other ones are stated in Annex no. 1 to Act no. 461/2003 Coll. on social insurance.

**Table 2:** Chronology of progress in health care at work.

Period	Figure	Publication Title
-	Hippokrates (460–370 BC)	“The Father of Medicine” First Recorded Mention of Occupational Diseases
-	Titus Lucretius Carus (99–55 BC)	Drew attention to the short life of miners in connection with their work
-	Gaius Plinius Secundus (AD 23–79)	Observed toxicity in the mining and processing of zinc and sulfur
-	Marcus Valerius Martialis (AD 40–102)	Warned of the danger of work with sulfur and blindness in blacksmiths
-	Aelius Claudius Galenus (AD 129–217)	Mentioned the risks arising in copper sulfate extraction in Cyprus
1473	Ulrich Ellenbog (1435–1499)	Pamphlet on Lead and Mercury Poisoning Among Gold Miners
1534–38	Paracelsus (1493–1541)	On miners’ diseases (1534) “Dosis Facit Venenum” (1538)
1556	Georg Agricola (1494–1555)	“De Re Metallica libri XII” (postmorte)
1656	Samuel Stockhausen (1649–1656)	“Libellus de lithargyrii fumo noxio morbifico eiusque metallico frequentiori morbo, vulgo dicto Die Hüttenkatze” [101]
1700–13	Bernardino Ramazzini (1633–1714)	“De Morbis Artificum Diatriba” (1700 rev. 1713)
1753	James Lind (1716–1794)	Treatise of the Scurvy
1761	Giovanni Antonio Scopoli (1723–1788)	“De Hydroargyro Idriensi Tentamina on the symptoms of mercury poisoning among mercury miners”
1767	George Baker (1722–1809)	An Essay Concerning the Cause of the Endemial Colic of Devonshire [102]
1775	Percival Pott (1714–1788)	Chirurgical Observations Relative to the Cataract, the Polypus of the Nose, the Cancer of the Scrotum, the Different Kinds of Ruptures, and the Mortification of the Toes and Feet
1778	José Parés y Franqués (1720–1798)	Catastrofe Morboso de las Minas Mercuriales de la Villa de Almaden del Azogue
1832	Charles Turner Thackrah (1795–1833)	The Effects of Arts, Trades, and Professions: and of Civic States and Habits of Living, on Health and Longevity: with Suggestions for the Removal of Many of the Agents which Produce Disease, and Shorten and Duration of Life
1832	James Phillips Kay-Shuttleworth (1804–1877)	The Moral and Physical Condition of the Working Classes Employed in the Cotton Manufacture in Manchester
1833	Great Britain	Factories Act adopted to improve the conditions of children working in factories (textile industry)
1837	Benjamin William McCready (1813–1892)	On the Influence of Trades, Professions, and Occupations in the United States, in the Production of Disease
1842	Edwin Chadwick (1800–1890)	Into the Sanitary Condition of the Labouring Population of Great Britain
1844	Robert Peel (1788–1850)	Health and Morals of Apprentices Act 1802 Cotton Mills and Factories Act 1819 Factories Act 1844
1845	Frederick Engels (1820–1895)	The Condition of the Working Class in England
1848	Rudolf Virchow (1821–1902)	Report on the Typhus Epidemic in Upper Silesia
1850	Lemuel Shattuck (1793–1859)	Report of the Sanitary Commission of Massachusetts [103]
1866–73	William H. Sylvis (1828–1869)	Founder of the National Labor Union in the USA
1869	Massachusetts	Introduction the first State Bureau of Statistics of Labor
1878	Knights of Labor	Requests a federal act on occupational safety and health
1880	Great Britain	Initiates a law on compensation for workers paid by the employer
1886	George H. Ireland (1850–1916)	The Preventable Causes of Disease, Injury, and Death in American Manufactories and Workshops, and the Best Means and Appliances for Preventing and Avoiding Them
1902	Thomas Oliver (1853–1942)	Dangerous Trades: Dangerous Trades: The Historical, Social, and Legal, Aspects of Industrial Occupations as Affecting health [104]
1905–34	Thomas Morison Legge (1863–1932)	Industrial Anthrax (1905)
		Lead Poisoning and Lead Absorption: The Symptoms, Pathology and Prevention, with Special Reference to their Industrial Origin and an Account of the Principle Processes Involving Risk (1912)
		Chronic Benzol Poisoning (1919) [105]
		Industrial Diseases Under the Medieval Trade Guilds (1920) [106]
		Charles Turner Thackrah: A Pioneer in Industrial Hygiene (1920) [107]
		Industrial Maladies (1934) [108]
		A Historical Background of Industrial Hygiene (1946) [109]
1910	Crystal Eastman (1881–1928)	Work-accidents and the Law [110]
1910	John Andrews Fitch (1881–1959)	The Steel Workers [111]
1914	William Gilman Thompson (1856–1927)	The Occupational Diseases: Their Causation, Symptoms, Treatment and Prevention [112]
1919	Switzerland	11 April 1919 creation of the ILO—International Labour Organization
1918–43	Alice Hamilton (1869–1970)	A Study of Spastic Anemia in the Hands of Stonecutters [113] (1918)
		Industrial Poisons in the United States (1925)
		Industrial toxicology (1934 rev. 1949)
		Exploring the Dangerous Trades: The Autobiography of Alice Hamilton (1943)
1942–64	Wilhelm Carl Hueper (1894–1978)	Occupational Tumors and Allied Diseases (1942)
		Chemical Carcinogenesis and Cancers (1964)
1933	Robley D. Evans (1907–1995)	Radium Poisoning A Review of Present Knowledge [114]
1946	Harriet Louise Hardy (1906–1993)	Delayed Chemical Pneumonitis Occurring in Workers Exposed to Beryllium Compounds [115]
1948	Switzerland	7 April 1948 creation of the WHO—World Health Organization
1953–61	Robert A. Kehoe (1893–1992)	Experimental Studies on the Inhalation of Lead by Human Subjects (1953)
		Occupational Medicine and Public Health (1961)
1941–76	Leonard J. Goldwater (1903–1992)	Disturbances in the Blood Following Exposure to Benzol [116] (1941)
		Fifteen Years of Cardiac Work Classification (1959)
		Strengthening Environmental Standards [117] (1976)
1970	Washington DC	29 December 1970 creation of the NIOS—National Institute for Occupational Health and safety
1971	Washington DC	28 April 1971 creation of the OSHA—Occupational Health and safety Administration
1994	Spain	18 June 1994 creation of the EU-OSHA—European Agency for Occupational health and safety
1968–91	Irving Selikoff (1915–1992)	Asbestos Exposure, Smoking, and Neoplasia (1968)
		Decline in Death Rates among Asbestos Insulation Workers 1967–1986 Associated with Diminution of Work Exposure to Asbestos (1990)
		Associated with Diminution of Work Exposure to Asbestos (1990)
		Asbestos Disease—1990–2020: The Risks of Asbestos Risk Assessment (1991)
		Asbestos-Associated Deaths among Insulation Workers in the United States and Canada [118] (1991)
1951–97	Thomas F. Mancuso (1912–2004)	Occupational Cancer and other Health Hazards in a Chromate Plant: A Medical Appraisal. I. Lung Cancers in Chromate Workers. (1951)
		Occupational Cancer and other Health Hazards in a Chromate Plant: A Medical Appraisal. II. Clinical and Toxicologic Aspects. (1951)
		Radiation Exposures of Hanford Workers Dying from Cancer and other Causes (1977)
		Chromium as an industrial carcinogen: Part I. (1997)
		Chromium as an Industrial Carcinogen: Part II. Chromium in Human Tissues (1997)

**Table 3:** Comparison of occupational diseases reported in the last 32 years.

Item	1987–2002	2003–2019	Total	Decrease/Increase	% *
21–23 ^c^	144	33	177	−111	−77.08
22	2125	440	2565	−1685	−79.29
24–26	3750	766	4516	−2984	−79.57
28	2002	1289	3291	−713	−35.61
29	1970	3065	5035	+1095	+55.58
33–34	637	258	895	−379	−59.50
38	1684	513	2197	−1171	−69.54
46	(-)	37	37	(x)	(x)
Total	14,008	7037	21,025	−6971	−49.76

* (−) decrease or (+) increase expressed as a percentage over the second half of the period compared to 1987–2002; (c) cancers together; hyphen (-) the phenomenon did not occur; cross (x) he entry is not possible for logical reasons.

**Table 4:** Overview of indicators and subcategories.

Indicator	Category
Gender	Male, Female
Age category	up to 29 years, from 30 to 39 years, 40 to 49 years, 50 to 59 years old, 60 and over
Economic activity sector	Agriculture and Forestry (Sector 1)
	Mining and Quarrying (Sector 2),
	Industrial Production (Sector 3), Construction (Sector 4)

**Table 5:** Overview of ETS models.

Model	AIC	Model	AIC
ETS(M,M,N)	248.35	ETS(M,N,N)	254.86
ETS(M,Md,N)	248.14	ETS(A,N,N)	258.33
ETS(M,A,N)	254.72	ETS(A,A,N)	253.66
ETS(M,Ad,N)	253.92	ETS(A,Ad,N)	249.88

Note: M—multiplicative, A—Additive, N—None, Md—Multiplicative damped, Ad—Additive damped.

**Table 6:** Forecast and prediction interval over a period of 5 years.

Year	Forecast	80% Prediction Interval		95% Prediction Interval	
		Lower	Upper	Lower	Upper
2020	299.38	254.18	344.74	231.71	367.80
2021	290.63	246.54	334.08	223.35	357.01
2022	282.32	240.71	324.82	218.81	347.09
2023	274.44	233.58	316.49	212.41	336.98
2024	266.95	228.05	307.34	208.11	329.02

## Data Availability

Publicly available datasets were analyzed in this study. This data can be found here: http://www.nczisk.sk/en/Publications/Edition_Health_Statistics/Pages/Archive.aspx, accessed on 22 April 2021.
